# Mechanisms underlying extremely fast muscle $\dot{V}$O_2_ on‐kinetics in humans

**DOI:** 10.14814/phy2.13808

**Published:** 2018-08-28

**Authors:** Bernard Korzeniewski, Harry B. Rossiter, Jerzy A. Zoladz

**Affiliations:** ^1^ BioSimulation Center Kraków Poland; ^2^ Rehabilitation Clinical Trials Center Division of Pulmonary Critical Care Physiology and Medicine Los Angeles Biomedical Research Institute at Harbor‐UCLA Medical Center Torrance California; ^3^ Faculty of Biological Sciences University of Leeds Leeds United Kingdom; ^4^ Department of Muscle Physiology Chair of Physiology and Biochemistry Faculty of Rehabilitation University School of Physical Education Kraków Poland

**Keywords:** Computer model, oxygen uptake kinetics, physical exercise, physical training, skeletal muscle

## Abstract

The time constant of the primary phase of pulmonary $\dot{V}$O_2_ on‐kinetics (*τ*_p_), which reflects muscle $\dot{V}$O_2_ kinetics during moderate‐intensity exercise, is about 30 s in young healthy untrained individuals, while it can be as low as 8 s in endurance‐trained athletes. We aimed to determine the intramuscular factors that enable very low values of *t*_0.63_ to be achieved (analogous to *τ*_p_, *t*_0.63_ is the time to reach 63% of the $\dot{V}$O_2_ amplitude). A computer model of oxidative phosphorylation (OXPHOS) in skeletal muscle was used. Muscle t_0.63_ was near‐linearly proportional to the difference in phosphocreatine (PCr) concentration between rest and work (ΔPCr). Of the two main factors that determine t_0.63_, a huge increase in either OXPHOS activity (six‐ to eightfold) or each‐step activation (ESA) of OXPHOS intensity (>3‐fold) was needed to reduce muscle *t*_0.63_ from the reference value of 29 s (selected to represent young untrained subjects) to below 10 s (observed in athletes) when altered separately. On the other hand, the effect of a simultaneous increase of both OXPHOS activity and ESA intensity required only a twofold elevation of each to decrease *t*_0.63_ below 10 s. Of note, the dependence of *t*_0.63_ on OXPHOS activity and ESA intensity is hyperbolic, meaning that in trained individuals a large increase in OXPHOS activity and ESA intensity are required to elicit a small reduction in *τ*_p_. In summary, we postulate that the synergistic action of elevated OXPHOS activity and ESA intensity is responsible for extremely low *τ*_p_ (*t*_0.63_) observed in highly endurance‐trained athletes.

## Introduction

The exponential time constant *τ* of phase 2 of oxygen consumption ($\dot{V}$O_2_) on‐kinetics in skeletal muscle (i.e., the time to reach 63% of the $\dot{V}$O_2_ amplitude, termed *τ*_p_ or *t*_0.63_) is the fundamental parameter characterizing the muscle bioenergetic system (Grassi et al. 1996; Whipp and Rossiter 2005; Zoladz et al. 2014). During moderate‐intensity (i.e., below the lactate threshold) whole‐body exercise such as cycling, muscle $\dot{V}$O_2_ on‐kinetics is well reflected by the time constant of phase 2 pulmonary $\dot{V}$O_2_ on‐kinetics (*τ*_p_) (Grassi et al. 1996). In young healthy untrained individuals during moderate‐intensity exercise *τ*_p_ approximately ranges 25–40 s (Whipp and Rossiter 2005; Grassi et al. 2011; Zoladz et al. 2013), while in well‐trained endurance athletes it can be as low as 8 s (Barstow and Molé 1991; Jones and Koppo 2005; Zoladz et al. 2005). In patients with cardiopulmonary diseases, both or either oxygen delivery and muscle oxygen extraction may be compromised during the first minutes of exercise, with the result that pulmonary *τ*_p_ can exceed 60 s (Grassi et al. 2011). Pathologically slow kinetics is predictive of poor prognosis (Schalcher et al. 2003). Pulmonary phase 2 $\dot{V}$O_2_ kinetics is also slow in mitochondrial diseases and McArdle's disease (Grassi et al. 2009), where oxidative phosphorylation (OXPHOS) activity is compromised.

Several animal‐based and human experiments have supported the notion that muscle $\dot{V}$O_2_ on‐kinetics during moderate‐intensity exercise in healthy young subjects is not limited by convective or diffusive oxygen delivery (Grassi et al. 1998; Bangsbo et al. 2000; Grassi 2005; Nyberg et al. 2014; Richardson et al. 2015). Most of the available experimental evidence suggests that muscle $\dot{V}$O_2_ on‐kinetics is mainly controlled or limited by intramuscular factors related to metabolic activation (Grassi et al. 2011; Rossiter 2011; Wüst et al. 2011; Poole and Jones 2012). However, the factors determining the value of muscle *τ*_p_ are not fully understood.

Previous theoretical studies demonstrated that two main factors determine muscle $\dot{V}$O_2_ on‐kinetics: OXPHOS activity and each‐step activation (ESA) intensity (Korzeniewski and Zoladz 2004). ESA was proposed as the main mechanism responsible for the regulation of OXPHOS during work transitions in skeletal muscle and heart (Korzeniewski 1998, 2007, 2017; Zoladz et al. 2013, 2014; Korzeniewski and Rossiter 2015). This mechanism was first named “parallel activation” (Korzeniewski 1998), but afterwards renamed “each‐step activation” in order to avoid confusion with other mechanisms involving simultaneous activation of ATP usage and ATP supply (Korzeniewski 2007; Korzeniewski and Rossiter 2015). A parallel activation of ATP usage and ATP supply block in general was first proposed by Hochachka (1994). On the basis of theoretical studies, ESA is characterized by direct activation of all OXPHOS complexes (complex I, complex III, complex IV, ATP synthase, ATP/ADP carrier, and P_i_ carrier), the NADH supply block, and glycolysis, in parallel with the activation of ATP usage during rest‐to‐work or low‐to‐high work transitions.

These computational simulations showed that muscle $\dot{V}$O_2_ on‐kinetics, using values typical of healthy young subjects, could be reasonably predicted on the basis of the known biochemical properties and the difference between resting and exercising PCr concentration (ΔPCr) (Korzeniewski and Zoladz 2004). However, these simulations interrogated only a relatively narrow range of OXPHOS activity and ESA intensity, and only the independent effects of either OXPHOS activity or ESA intensity were studied; not the synergistic effect of both these factors acting simultaneously.

Muscle mitochondrial volume (density) is about twofold greater in highly trained individuals (e.g., $\dot{V}$O_2max_ 70–80 mL min^−1^ kg^−1^) compared to average young healthy untrained individuals ($\dot{V}$O_2max_ 40–50 mL min^−1^ kg^−1^) (Hoppeler et al. 1973). A more recent study (Larsen et al. 2012) found a ~3‐fold range of muscle mitochondrial volume (density) by transmission electron microscopy, OXPHOS complex concentration by protein quantification, and OXPHOS activity by respirometry, in a group of individuals with $\dot{V}$O_2max_ ranging 30–72 mL min^−1^ kg^−1^.

We therefore studied the effect of large changes in OXPHOS activity and ESA intensity on ΔPCr and muscle *t*_0.63_ to determine the skeletal muscle bioenergetic system characteristics required to elicit very low *τ*_p_ values observed in athletes. Both the separate effect of each factor and the synergistic effect of each acting simultaneously were analyzed. It was hypothesized that a huge increase in either OXPHOS activity or ESA intensity separately is necessary to decrease muscle *t*_0.63_ below 10 s, while only a moderate increase is sufficient when the magnitudes of both factors are elevated simultaneously. It was also hypothesized that the dependence of muscle *t*_0.63_ on OXPHOS activity (kOX) and ESA intensity (*A*_OX_) is hyperbolic rather than linear, and therefore *t*_0.63_ would be much more sensitive to changes in kOX and *A*_OX_ at low kOX and *A*_OX_ values, than at high kOX and *A*_OX_ values.

## Theoretical Methods

### Computer model

The well‐tested computer model of OXPHOS and the entire bioenergetic system in intact skeletal muscle was used in the simulations carried out in this study (Korzeniewski and Zoladz 2001; Korzeniewski and Liguzinski 2004). This model comprises explicitly NADH supply block (TCA cycle, fatty‐acid *β*‐oxidation, MAS, etc.), particular OXPHOS complexes (complex I, complex III, complex IV, ATP synthase, ATP/ADP carrier, and *P*_i_ carrier), proton leak through the inner mitochondrial membrane, glycolysis (aerobic and anaerobic), ATP usage, creatine kinase (CK), and proton efflux/influx to/from blood. The complete description of the model is located on the web site: http://awe.mol.uj.edu.pl/~benio/. It can be also found in Korzeniewski and Zoladz (2001), supplemented with modifications in Korzeniewski and Liguzinski (2004) and Korzeniewski and Rossiter (2015).

### Computer simulations

The two main parameters used in this study can be defined as follows. Relative OXPHOS activity (kOX) is the unitless basal value of the rate constants of all OXPHOS complexes expressed relative to 1 in the reference (standard) state. kOX is the relative OXPHOS activity in the absence of ESA. ESA intensity (*A*_OX_) is the unitless fold increase in the values of the rate constants of all OXPHOS complexes during rest‐to‐work transition equal to 4.5 in the reference (standard) state. Therefore, kOX is the relative OXPHOS activity at rest and kOX × *A*_OX_ is the relative OXPHOS activity during work. Neither kOX, constituting the relative rate constant of OXPHOS at rest, nor kOX × *A*_OX_, representing the relative rate constant of OXPHOS during work, is equivalent to the absolute flux of muscle oxygen consumption ($\dot{V}$O_2_, or its equivalent ATP synthesis flux) expressed in mmol/L min^−1^. $\dot{V}$O_2_ depends not only on absolute OXPHOS activity (rate constant) but also on metabolite concentrations, for example, of ADP and *P*_i_. kOX is, of course, related to mitochondrial density as well as to the concentration and activity of OXPHOS complex enzymes.

Moderate exercise was analyzed in this study, in which the activity of ATP usage was 30 times higher than at rest. This rate is anticipated to be approximately equivalent to about 30–35% of the muscle $\dot{V}$O_2max_ measured during cycle ergometry. This corresponds to about 40% of the pulmonary $\dot{V}$O_2max_ during cycling. The exact work intensity applied is of minor importance, as *τ*_p_ depends little on work intensity in constant‐power exercise (Poole and Jones 2005). The reference state used for the simulations corresponded to that of young healthy untrained people (with pulmonary $\dot{V}$O_2max_ = 40–50 mL min^−1^ kg^−1^ and *τ*_p_ = 29.2 s) (e.g., Hoppeler et al. 1973; Rossiter 2011). The standard OXPHOS activity appearing in the model (the reference state) is indicated as “kOX * 1”, that is, onefold of the standard OXPHOS activity increase. In order to obtain muscle *t*_0.63_ equal to about 30 s during moderate work for the reference state, an ESA intensity (*A*_OX_) equal to 4.5 was required. In this reference state the muscle $\dot{V}$O_2_ = 3.62 mmol/L min^−1^, resting PCr = 27.74 mmol/L, ΔPCr = 10.80 mmol/L, and muscle *t*_0.63_ = 29.2 s.

In subsequent simulations either OXPHOS activity or ESA intensity was changed separately, or both were changed simultaneously. An *n*‐fold increase in the resting (without ESA) OXPHOS activity (kOX*n) was equivalent to an *n*‐fold increase in resting rate constants of all OXPHOS complexes and NADH supply (k_C1_, k_C3_, k_C4_, k_SN_, k_EX_, k_PI_, and k_DH_). The resting OXPHOS activity was either increased (*n* > 1) or decreased (*n* < 1) in relation to the reference state. Proton leak activity was not changed, as it was assumed that the increase in the proton leak activity related to the twofold greater mitochondrial volume between the reference state (young healthy untrained) and the endurance‐trained state (with pulmonary $\dot{V}$O_2max_ above 70 mL min^−1^ kg^−1^ and *τ*_p_ < 10 s) is compensated by a training‐induced twofold decrease in proton leak intensity per mitochondrial volume. This assumption is partly based on experimental data illustrating the magnitude of the endurance training‐induced mitochondrial biogenesis and oxidative capacity, was accompanied by a decrease in mitochondrial uncoupling, including uncoupling protein‐mediated proton leak studied in rat skeletal muscle mitochondria (Zoladz et al. 2016). A particular value of ESA intensity (*A*_OX_) meant that the rate constants of all OXPHOS complexes and NADH supply (k_C1_, k_C3_, k_C4_, k_SN_, k_EX_, k_PI_, and k_DH_) were elevated *A*_OX_ times during rest‐to‐work transitions. *A*_OX_ was either increased (*A*_OX_ > 4.5) or decreased (*A*_OX_ < 4.5) in relation to the reference state.

## Results of Simulations

This is a purely theoretical work and does not involve experiments on humans or animals.

**Figure 1:**
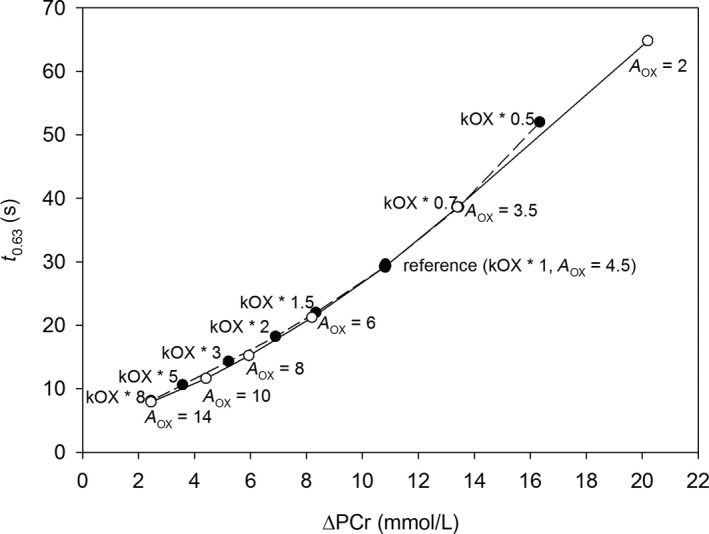
Simulated dependence of the time to reach 63% of the muscle $\dot{V}$O_2_ amplitude (*t*_0.63_) on the difference between PCr during work and rest (ΔPCr). ΔPCr was modified either by an increase/decrease in resting OXPHOS activity (kOX) without a change in ESA intensity (*A*_OX_), or by an increase/decrease in *A*_OX_ in relation to the reference state (young, healthy, untrained individuals).

Figure 1 demonstrates that muscle *t*_0.63_ had a near‐linear dependence on the difference between PCr at rest and during exercise (ΔPCr). This relationship was independent of whether ΔPCr was changed through a change in the resting OXPHOS activity (kOX), that is, without ESA, or through a change in ESA intensity (*A*_OX_). However, when the resting OXPHOS activity and ESA intensity were modified separately, a huge increase in these parameter values was necessary in order to diminish muscle *t*_0.63_ significantly from the reference state value of 29.2 s. Specifically, an approximate sixfold increase in resting OXPHOS activity was required in order to decrease muscle *t*_0.63_ to 10 s and an eightfold increase was necessary to reach muscle *t*_0.63_ = 8 s. Alternatively, it was necessary to increase ESA intensity 3.1‐fold (from *A*_OX_ = 4.5 to *A*_OX_ = 14) to diminish muscle *t*_0.63_ to 8 s.

Both resting (without ESA) OXPHOS activity and ESA intensity affect the overall OXPHOS activity during muscle work. However, resting OXPHOS activity affects the resting PCr concentration, while ESA intensity does not: *A*_OX_ acts only during muscle work. This is why the former must be elevated eightfold and the latter only 3.1‐fold in order to evoke the same decrease in muscle *t*_0.63_ (to 8 s). An increase in resting OXPHOS activity demands an elevation in resting PCr concentration. This in turn requires an increased ΔPCr and thus slows down muscle *t*_0.63_. Therefore, a relatively high increase in the resting OXPHOS activity kOX is necessary in order to diminish muscle *t*_0.63_ below 10 s. This effect is absent in case of increasing ESA intensity, and therefore adjusting ESA intensity was more effective in decreasing *t*_0.63_ than an adjustment in resting (without ESA) OXPHOS activity.

**Figure 2:**
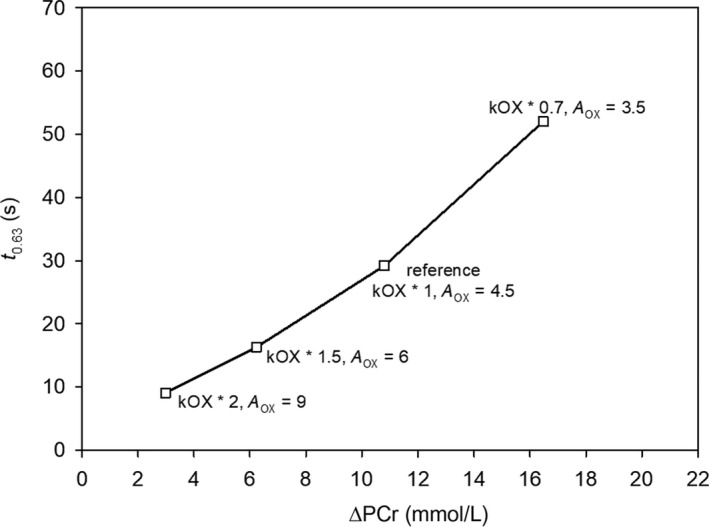
The simulated synergistic effect of a simultaneous increase/decrease in both resting (without ESA) OXPHOS activity and ESA intensity on the relationship between muscle *t*_0.63_ and ΔPCr. Resting OXPHOS activity (kOX) and ESA intensity (*A*_OX_) are changed in parallel in relation to the reference state (young, healthy, untrained individuals).

The requirement for a huge increase in resting (without ESA) OXPHOS activity and, to a lesser extent, ESA intensity could be avoided by using the synergistic effect of a simultaneous increase in the magnitude of both factors together. This is demonstrated in Figure 2. A parallel twofold increase in resting (without ESA) OXPHOS activity (kOX) and ESA intensity (*A*_OX_) (increasing *A*_OX_ from 4.5 to 9) leads to a decrease of muscle *t*_0.63_ from 29 s in the reference state to 9 s in the “activated” state. Therefore, the synergistic effect of increasing both resting OXPHOS activity and ESA intensity was very effective in decreasing the *t*_0.63_ of muscle $\dot{V}$O_2_.

**Figure 3:**
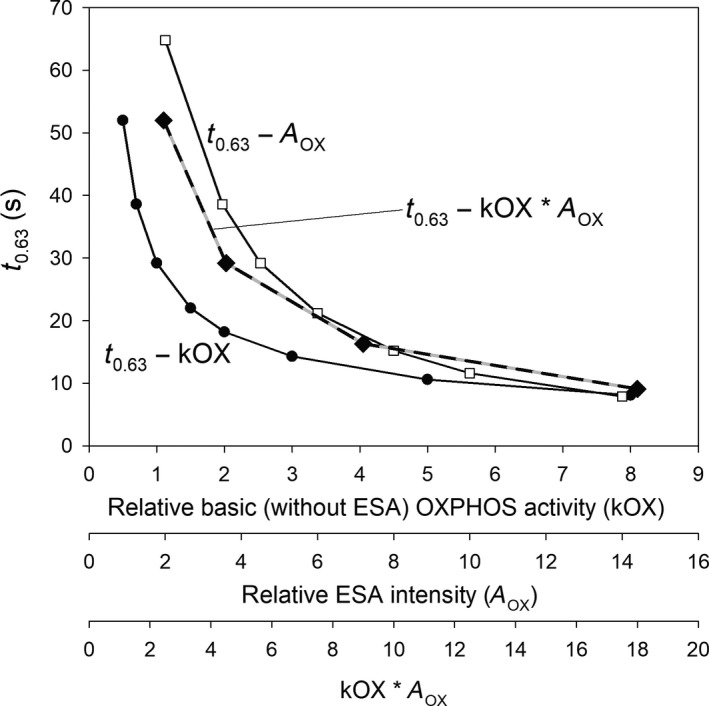
The simulated dependence of muscle *t*_0.63_ on relative resting (without ESA) OXPHOS activity (kOX; filled circle), ESA intensity (*A*_OX_; open square), and the product of kOX and *A*_OX_ (kOX*A_OX_; filled diamond). These dependencies are extracted from Figures 1 and 2.

Simulated dependence of muscle *t*_0.63_ on relative reference OXPHOS activity (kOX), relative ESA intensity (*A*_OX_), and the product of kOX and *A*_OX_ is essentially hyperbolic. This is demonstrated in Figure 3. These dependencies are extracted from Figures 1 and 2.

**Figure 4:**
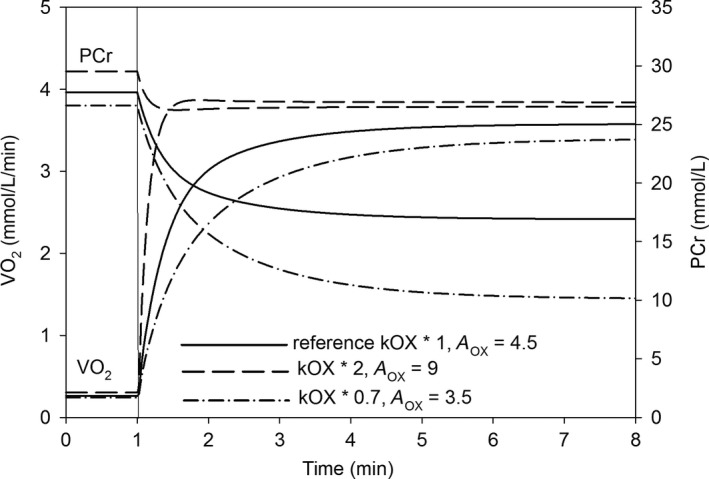
Simulated time course of muscle $\dot{V}$O_2_ and PCr during a rest‐to‐moderate work transition for the reference state (solid line), parallel twofold increase in the resting (without ESA) OXPHOS activity (kOX) and ESA intensity (*A*_OX_) (dashed line), and parallel decrease in the resting OXPHOS activity (to 70% of the reference value) and in ESA intensity (to 78% of the reference value) (dash‐dot line). The reference values are for young, healthy, untrained individuals (pulmonary $\dot{V}$O_2max_ = 40–50 mL min^−1^ kg^−1^ and *τ*_p_ = 29.2 s)

The fact that ΔPCr between rest and work is strictly related to muscle *t*_0.63_ is illustrated in Figure 4. One can see that when both resting OXPHOS activity and ESA intensity are elevated in relation to the reference state, ΔPCr is much smaller and $\dot{V}$O_2_ kinetics is speeded compared with the reference state. On the other hand, when the magnitudes of both factors are decreased, ΔPCr is significantly greater and $\dot{V}$O_2_ kinetics is significantly slowed in relation to the reference state.

## Discussion

This theoretical intramuscular study demonstrates that: (1) both an increase in resting (without ESA) OXPHOS activity and ESA intensity can significantly accelerate the muscle $\dot{V}$O_2_ on‐kinetics (reduce *t*_0.63_); (2) muscle *t*_0.63_ is near‐linearly proportional to the difference in the PCr concentration between work and rest (ΔPCr); (3) the muscle *t*_0.63_−ΔPCr dependence is identical regardless of whether ΔPCr is changed through an increase in OXPHOS activity or through an increase in ESA intensity; (4) when the magnitudes of OXPHOS activity or in ESA intensity are increased separately, a huge increase in resting OXPHOS activity (six‐ to eightfold) or ESA intensity (>3‐fold) is necessary to decrease the muscle *t*_0.63_ from 29 s in the reference state (young healthy individuals) to 8 s (as observed in the extreme endurance‐trained state); (5) the required increasing resting OXPHOS activity of >6‐fold to decrease the muscle *t*_0.63_ to <10 s exceeds greatly the observed increases with endurance training in muscle mitochondrial volume (density) of two‐ to threefold; and (6) a synergistic effect of a simultaneous increase in resting OXPHOS activity and ESA intensity together requires only twofold increases in the magnitudes of these factors to decrease *t*_0.63_ from 29 to 8 s. Therefore, we postulate that the very short *τ*_p_ (*t*_0.63_) of the muscle and pulmonary $\dot{V}$O_2_ on‐kinetics encountered in very highly endurance‐trained athletes are due to a high mitochondrial volume (density) related to high (resting) OXPHOS activity as well as a high ESA intensity. Each can result from both genetic factors (interindividual differences) and endurance training.

It is demonstrated that the dependence of the muscle *t*_0.63_ on OXPHOS activity and ESA intensity is essentially hyperbolic, as shown in Figure 3. A hyperbolic relationship between *t*_0.63_ and whole‐body $\dot{V}$O_2max_ in different mammal species was previously described (Poole and Jones 2012). Here, we show that, in the context of muscle $\dot{V}$O_2_, independent of oxygen delivery, this hyperbolic relationship is anticipated with training‐induced increases in OXPHOS activity and ESA intensity alone, even in the absence of increased mitochondrial volume (density). This implies that a training‐induced increase in mitochondrial biogenesis and/or ESA intensity above the reference state would decrease *τ*_p_ only moderately (e.g., Zoladz et al. 2013), while a decrease in OXPHOS activity and/or ESA intensity, related to, for example, muscular deconditioning, enzyme deficiencies, or mitochondrial diseases, can lengthen *τ*_p_ very significantly. This theoretical prediction confirms experimental data from single frog muscle cells over a wide range of OXPHOS capacities (Wüst et al. 2013) and from endurance‐trained humans (Jones and Koppo 2005) or humans with metabolic mitochondrial myopathies (Grassi et al. 2009).

Mitochondrial volume (density) is about two‐ to threefold different between untrained individuals with pulmonary $\dot{V}$O_2max_ of ~30 mL min^−1^ kg^−1^ (Larsen et al. 2012), average young healthy individuals within the reference range used for this study (40–50 mL min^−1^ kg^−1^; Hoppeler et al. 1973), and after endurance training or in elite athletes with pulmonary $\dot{V}$O_2max_ 70–80 mL min^−1^ kg^−1^ (Larsen et al. 2012). Zoladz et al. (2013) showed, based on muscle biopsy of the vastus lateralis, that moderate‐intensity endurance training (85% of the total training performed below the LT, in this case ~50% $\dot{V}$O_2max_, and only 15% at midway between the lactate threshold and $\dot{V}$O_2max_), lasting 5 weeks (four sessions of cycling per week; 40 min per session), significantly accelerates the primary phase of the pulmonary $\dot{V}$O_2_ on‐kinetics (by about 25%) in humans in the absence of any changes in the markers of mitochondrial biogenesis such as the level of peroxisome proliferator‐activated receptor *γ* coactivator 1*α* (PGC1 *α*), mitochondrial DNA copy number, cytochrome *c*, and cytochrome oxidase subunit I contents. This presupposes that the alterations in the muscle biopsy reflect the action of the endurance training on the activated muscle during moderate‐intensity cycling. If it does, it would support the postulate, based on computer simulations, that the training‐induced acceleration of the $\dot{V}$O_2_ on‐kinetics observed during moderate‐intensity cycling, accompanied by no signs of elevated OXPHOS activity, could be explained by a training‐induced intensification of ESA (Zoladz et al. 2013). In other words, physical training first induces the increase in ESA intensity (*A*_OX_), which is followed by mitochondrial biogenesis (providing an increase in OXPHOS activity, kOX). Similarly, prolonged moderate‐intensity endurance training, lasting 20 weeks (four sessions per week; 40 min per session), which speeded phase 2 pulmonary $\dot{V}$O_2_ on‐kinetics by about 19% was accompanied by no significant changes in the maximal COX activity in the muscle vastus lateralis (Zoladz et al. 2014). It was concluded based on the theoretical studies described therein (Zoladz et al. 2014) that the most likely explanation was a training‐induced intensification of each‐step parallel activation (ESA) of OXPHOS that occurred before elevated mitochondrial biogenesis.

Some studies have postulated a significant role of O_2_ delivery (Murias et al. 2014) and/or of the uniformity of matching between intramuscular O_2_ delivery and O_2_ utilization (Koga et al. 2014) in determining the rate of $\dot{V}$O_2_ on‐kinetics. However, several experimental studies in animal models and humans have failed to show a limitation of oxygen transport to muscle for $\dot{V}$O_2_ on‐kinetics during transition to exercise, at least during exercise of moderate intensity (Grassi et al. 1998; Bangsbo et al. 2000; Grassi 2005; Nyberg et al. 2014; Richardson et al. 2015).

In our study, the rate of the muscle $\dot{V}$O_2_ on‐kinetics during rest‐to‐work transition is expressed by the time to reach 63% of the $\dot{V}$O_2_ amplitude (*t*_0.63_). This parameter does not require the $\dot{V}$O_2_ on‐kinetics to be strictly exponential, although the simulated $\dot{V}$O_2_ on‐kinetics is near exponential (Fig. 4). It was argued previously (Korzeniewski and Zoladz 2006) that the behavior of the muscle $\dot{V}$O_2_ time course during the rest‐to‐work transition is not perfectly exponential, as it is slightly faster at the very beginning of exercise due to ESA. Such a very fast increase in $\dot{V}$O_2_ during first seconds of exercise can be seen in some animal (Zoladz et al. 2008; Wüst et al. 2011) and human studies (Chung et al. 2005), which confirms the ESA hypothesis. However, this effect is complex to interpret in pulmonary $\dot{V}$O_2_ because of the action of the phase 1 on‐kinetics kinetics, which is manifest at the lung and not at the muscle. On the other hand, Hogan (2001) encountered a lag in the fall of intracellular PO_2_ after the onset of contractions in single *Xenopus* muscle fibers, suggesting a lag in the $\dot{V}$O_2_ increase. It has been demonstrated in simulation and by direct experiment that the phase 2 pulmonary $\dot{V}$O_2_ time course also deviates from a “pure” exponential response, albeit very subtly (Benson et al. 2013, 2017). Therefore, the muscle *t*_0.63_ variable reported here is very closely related, but not strictly identical, to the frequently used parameter describing the pulmonary $\dot{V}$O_2_ on‐kinetics, called time constant of the phase 2 (or primary phase) of this kinetics (*τ*_p_) (Barstow and Molé 1991; Scheuermann et al. 2001; Whipp and Rossiter 2005). Pulmonary $\dot{V}$O_2_ kinetics can deviate significantly from the muscle $\dot{V}$O_2_ kinetic response due to the influence of the dynamics of the circulation and the intervening venous blood and pulmonary gas volumes between the muscle cell and the pulmonary $\dot{V}$O_2_ measurement (Benson et al. 2013). However, it should be stressed that any difference between *t*_0.63_ and *τ* of muscle $\dot{V}$O_2_ in our simulations is disappearingly small and of little functional importance.

Muscle *t*_0.63_ is nearly linearly proportional to ΔPCr between work and rest, as demonstrated in Figure 1. This is in agreement with the results of the experiments by Barstow et al. (1994) and Phillips et al. (1995). Therefore, the rate of the $\dot{V}$O_2_ on‐kinetics reflects the magnitude of disturbances in muscle metabolic stability during rest‐to‐work transition (compare also Fig. 4). The extremely fast pulmonary $\dot{V}$O_2_ on‐kinetics (*τ*_p_ < 10 s) observed in top class endurance athletes (Barstow and Molé 1991; Jones and Koppo 2005; Zoladz et al. 2005), reflecting very high metabolic stability (Korzeniewski and Zoladz 2004), should be considered as a sign of very high resistance to fatigue during high‐intensity exercise (Murgatroyd et al. 2011; for review see Grassi et al. 2015; Keir et al.*,* 2016).

A moderate work intensity (30–35% of the muscle $\dot{V}$O_2max_, about 40% of the pulmonary $\dot{V}$O_2max_) was used in order to avoid the slow component of the $\dot{V}$O_2_ on‐kinetics. There is considerable evidence to support that O_2_ delivery is not limiting at this exercise intensity. In constant‐power exercise pulmonary *τ*_p_ depends little on work intensity: pulmonary *τ*_p_ increases on average by about 20% between moderate and heavy exercise (see Poole and Jones 2005; for review of over 20 experimental studies). Theoretical studies, concerning muscle *τ*_p_ (*t*_0.63_), confirmed these experimental findings (Korzeniewski 2018). Computer simulations predicted that muscle *t*_0.63_ was approximately constant for different work intensities in constant‐power exercise, and decreased noticeably only at lowest work intensities.

Of course, this theoretical study and the computer model used have several limitations. Every model constitutes, at best, only an approximation and simplification of the complex reality. We cannot prove that the mechanism proposed by us is entirely responsible for the extremely fast $\dot{V}$O_2_ on‐kinetics in top athletes, rather we provide a quantitative argument for a plausible explanation. The role of other factors, for instance, limitations of O_2_ transport and diffusion, cannot be excluded, although they seem not to be important during moderate exercise in healthy individuals. The estimation of some variables is only approximate, for example, differences in mitochondrial volume and OXPHOS activity between extreme individuals and reference individuals. Nevertheless, these restrictions do not seem to change the general conclusions of our simulations.

## Conclusions

Based on our theoretical study we postulate that the very short (<10 s) time constant (*τ*_p_ ~*t*_0.63_) of the primary phase of the pulmonary and muscle $\dot{V}$O_2_ on‐kinetics, encountered in highly endurance‐trained athletes, representing very high metabolic stability during exercise, results from the synergistic action of a high resting (without ESA) OXPHOS activity related to high mitochondrial volume (density) and high each‐step activation (ESA) of OXPHOS intensity. The dependence of muscle *τ*_p_ on OXPHOS activity and/or ESA intensity is hyperbolic. For this reason, it is difficult to further reduce *τ*_p_ by training in well‐trained individuals, while *τ*_p_ is greatly lengthened when OXPHOS activity is compromised in mitochondrial diseases.

## Conflict of Interest

None declared.
